# Combinatorial metabolic engineering of industrial *Gluconobacter oxydans* DSM2343 for boosting 5-keto-D-gluconic acid accumulation

**DOI:** 10.1186/s12896-016-0272-y

**Published:** 2016-05-17

**Authors:** Jianfeng Yuan, Mianbin Wu, Jianping Lin, Lirong Yang

**Affiliations:** Key Laboratory of Biomass Chemical Engineering of the Ministry of Education,College of Chemical and Biological Engineering, Zhejiang University, Hangzhou, 310027 China

**Keywords:** 5-keto-D-gluconate, L-(+)-tartaric acid, Pyrroloquinoline quinone, Respiratory chain, Fed-batch fermentation

## Abstract

**Background:**

L-(+)-tartaric acid (L-TA) is an important organic acid, which is produced from the cream of tartar or stereospecific hydrolysis of the *cis*-epoxysuccinate. The former method is limited by the availability of raw material and the latter is dependent on the petrochemical material. Thus, new processes for the economical preparation of L-TA from carbohydrate or renewable resource would be much more attractive. Production of 5-keto-D-gluconate (5-KGA) from glucose by *Gluconobacter oxydans* is the first step to produce L-TA. The aim of this work is to enhance 5-KGA accumulation using combinatorial metabolic engineering strategies in *G. oxydans*. The *sldAB* gene, encoding sorbitol dehydrogenase, was overexpressed in an industrial strain *G. oxydans* ZJU2 under a carefully selected promoter, P_0169_. To enhance the efficiency of the oxidation by *sldAB*, the coenzyme pyrroloquinoline quinone (PQQ) and respiratory chain were engineered. Besides, the role in *sldAB* overexpression, coenzyme and respiratory chain engineering and their subsequent effects on 5-KGA production were investigated.

**Results:**

An efficient, stable recombinant strain was constructed, whereas the 5-KGA production could be enhanced. By self-overexpressing the *sldAB* gene in *G. oxydans* ZJU2 under the constitutive promoter P_0169_, the resulting strain, *G. oxydans* ZJU3, produced 122.48 ± 0.41 g/L of 5-KGA. Furthermore, through the coenzyme and respiratory chain engineering, the titer and productivity of 5-KGA reached 144.52 ± 2.94 g/L and 2.26 g/(L · h), respectively, in a 15 L fermenter. It could be further improved the 5-KGA titer by 12.10 % through the fed-batch fermentation under the pH shift and dissolved oxygen tension (DOT) control condition, obtained 162 ± 2.12 g/L with the productivity of 2.53 g/(L · h) within 64 h.

**Conclusions:**

The 5-KGA production could be significantly enhanced with the combinatorial metabolic engineering strategy in *Gluconobacter* strain, including *sldAB* overexpression, coenzyme and respiratory chain engineering. Fed-batch fermentation could further enlarge the positive effect and increase the 5-KGA production. All of these demonstrated that the robust recombinant strain can efficiently produce 5-KGA in larger scale to fulfill the industrial production of L-TA from 5-KGA.

## Background

L-(+)-tartaric acid (L-TA), an important naturally existing hydroxyl carboxylic acid, is mainly used as antioxidant in food industry, as a chiral reagent in organic synthesis, as an acidic reducing agent in the textile industry and in galvanochemistry [1–3]. It is also an alternative to citric acid as an acidulant in food additives for its superior organoleptic properties [4]. Currently, the L-TA that is commercially available is produced exclusively through the stereospecific hydrolysis of *cis*-epoxysuccinate [5–7]. In this process, the reaction is catalyzed by the *cis*-epoxysuccinate hydrolase from *Rhodococcus rhodochrous*, *Nocardia tartaricans*, *Corynebacterium* sp. or *Pseudomonas* sp. [7]. However, *cis*-epoxysuccinate is derived from petrochemical-based precursor maleic anhydride, and this limits the production of L-TA. Therefore, development of the sustainable alternative solution for L-TA manufacture has recently attracted increasing attention.

A promising route has been employed for the production of L-TA by sequential whole-cell catalyzed oxidation and chemical catalysis, in which the glucose was first biologically converted to 5-keto-D-gluconate (5-KGA) by *Gluconobacter oxydans*, then to L-TA in the presence of ammonium vanadate as a trace element [4, 8]. Thus, the strategy towards an efficient synthetic route to L-TA was to optimize the enzymatic production of 5-KGA [4, 9].

**Fig. 1:**
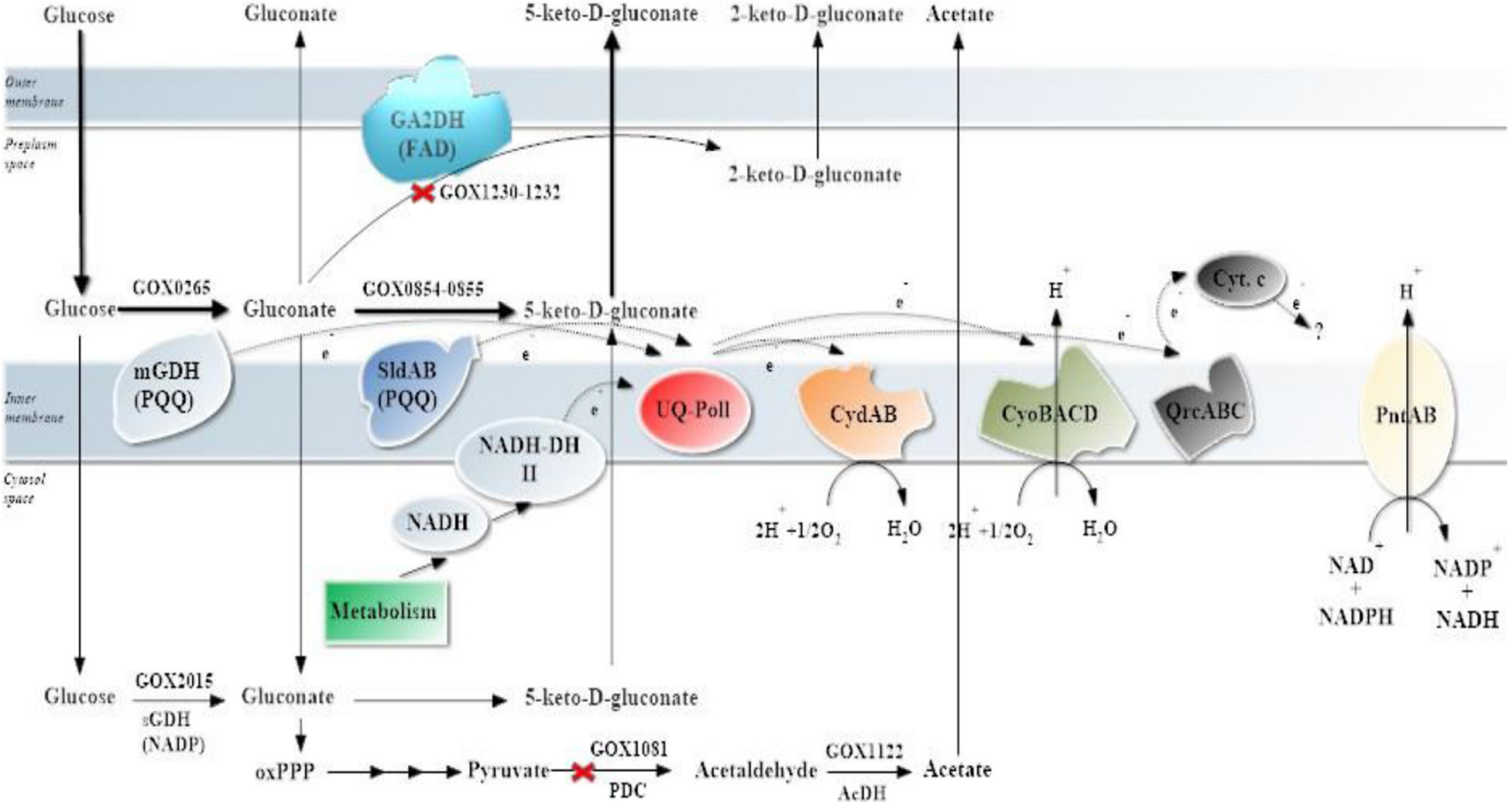
Scheme of glucose metabolism and the respiratory chain in *Gluconobacter oxydans* DSM2343. Abbreviations: mGDH, membrane-bound PQQ-dependent glucose dehydrogenase; SldAB, membrane-bound PQQ-dependent sorbitol dehydrogenase; GA2DH, membrane-bound FAD-dependent gluconate 2-dehydrogenase; CydAB, cytochrome *bd* oxidase; CyoBACD, cytochrome *bo*_3_ oxidase; QrcABC, cytochrome *bc*_1_ complex; PntAB, transhydrogenase. the red-cross means the metabolic pathway cut off

Through genomic analyses, the genome sequence of *G. oxydans* 621H has been published, which leads to new insights into its metabolic pathway [10]. The essential genetic elements related to 5-KGA metabolism have been systematically identified (Fig. 1). The membrane-bound glucose dehydrogenase (mGDH, GOX0265), a quinoprotein containing pyrroloquinoline quinone (PQQ), oxidizes D-glucose to D-glucono-δ-lactone, which is subsequently converted to gluconic acid (GA) spontaneously or by gluconolactonase [11–13]. GA can be further oxidized to 5-KGA or 2-keto-D-gluconate (2-KGA) by PQQ-dependent sorbitol dehydrogenase (SLDH, GOX0854-0855) or FAD-dependent gluconate 2-dehydrogenase (GA2DH, GOX1230-1232), which transfer electrons from glucose to the respiratory chain ubiquinone and then to terminal ubiquinol oxidases to generate the proton motive force [10, 14]. PQQ, heme *c* or FAD serve as prosthetic groups [15, 16].

So far several attempts have been made to increase 5-KGA production, such as overexpression of gluconate: NADP 5-oxidoreductase [9], inactivation of GA2DH [3, 17] and overexpression of SLDH [18, 19] and optimization of the media conditions [20]. The highest yield achieved of 5-KGA was 240–295 mM (about 60 g/L) in a batch fermentation over periods as long as 72 h with a productivity of 0.83 g/(L · h) [18]. However, the methods used are individual engineering approaches and the efficiency of 5-KGA production was unsatisfactory, especially there was a large amount of residual GA left at the end of the biotransformation process.

In early work, we constructed the recombinant strain *G. oxydans* ZJU2, in which the GOX1231 and GOX1081 genes were markerless deleted [21]. The engineering of 2-KGA and acetic acid metabolism pathway could enhanced the 5-KGA production, but the titer and productivity of 5-KGA was unsatisfied. Hence, we set out to develop an efficient *Gluconobacter* cell factory to facilitate 5-KGA biosynthesis using combinatorial strategies. The *sldAB* gene, encoding the SLDH, was plasmid-based overexpressed with a strong promoter, P_0169_ [22]. Inspired by the mechanism from most of the *Gluconobacter*, it is proposed that the cofactor PQQ and respiratory chain engineering could be enhanced efficiency of the membrane-bound quinoaproteins. As a result, the genes involved with the PQQ cluster [23, 24] and terminal ubiquinol cytochrome *bo*_3_ oxidase [25] were fused expression. The related specific enzyme activity, H^+^/O ratio and 5-KGA titer were investigated. Base on the pH shift and dissolved oxygen tension (DOT) control, the robust *G. oxydans* cells were more facilitated 5-KGA accumulation by the fed-batch fermentation. This study represents combinatorial engineering approaches collectively increased the titer of 5-KGA in the *G. oxydans*, which can provide insights into devising engineering strategies to improve the object production.

## Results and discussion

### Promoter selection in *G. oxydans* strain

**Fig. 2:**
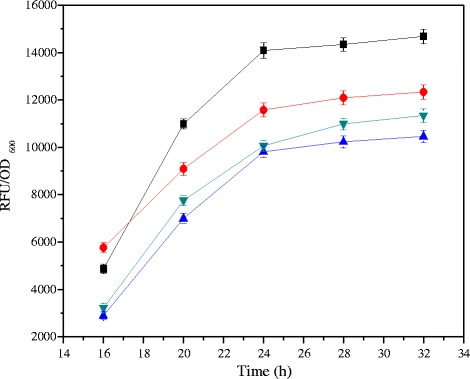
Expression of green fluorescent protein in *G. oxydans* DSM2343 under the control of different promoter. The whole cell relative fluorescence unit (RFU) are the averages of three different experiments divided by the cell density at 600 nm. ■ P_0169_ promoter,  P_0264_ promoter,  P_0452_ promoter,  P_tufB_ promoter

Increasing interest in *Gluconobacter* has led to investigations on strain improvement. The well characterized promoter is a prerequisite to the understanding of gene expression. So far, a few studies concerning the isolation and characterization of *G. oxydans* promoters, such as P_0169_ [22], P_tufB_ [26], P_0264_ [27], and P_0452_ [27], have been reported. However, which one is more suit for gene expression in *G. oxydans* is inconclusive. To select the appropriate promoter for gene expression in *G. oxydans*, we generated the different promoters in front of a *gfp* report gene in a pBBR1MCS5 vector. The promoter activity was indirectly determined by measuring the whole cell fluorescence intensity (RFU/OD_600_). As shown in Fig. 2, the RFU/OD_600_ presented a linear increase with the cell growth until 24 h. Green fluorescent protein (GFP) contains a fluorescent cyclic tri-peptide, and oxygen is required for the final oxidation of the mature, cyclized fluorophore of GFP [22]. For this reason, the RFU was impacted by the level of the DO, which explained that the RFU/OD_600_ remained stable after 24 h. However, among this four different promoters, the cells expressing *gfp* under regulation of P_0169_ promoter exhibited outstanding effect in the *G. oxydans*. The observation elucidated that the P_0169_ promoter was evidently recognized by *G. oxydans* and could reliably drive heterologous gene expression in *G. oxydans*.

### Enzyme activity and relative transcriptional level of *sldAB* overexpression strain

The glucose metabolism of the mutant *G. oxydans* ZJU2 strain showed the positive effect on the 5-KGA production [21], but it remained low productivity of 5-KGA. To boost the 5-KGA production, the *sldAB* gene was plasmid-based overexpressed in *G. oxydans* ZJU2 under the control of the selected P_0169_ promoter, generating *G. oxydans* ZJU3. The enzyme activity of SLDH of *G. oxydans* ZJU3 toward GA and the mRNA abundance of *sldAB* were investigated (Table 1). The SLDH activity was 2.55 ± 0.04 U/mg protein, which was more 3-fold higher than that of the reference strain. The *sldAB* expression data obtained were normalized to the transcriptional level of the 16S RNA. *G. oxydans* ZJU3 achieved the relative transcriptional level of 4.12 ± 0.04, which was 4-fold higher than those of the control. The results showed that the specific enzyme activities and transcription of the *sldAB* gene in *G. oxydans* could be markedly enhanced by *sldAB* overexpressed under the control of the selected P_0169_ promoter. In the literature [26], the stability and transcriptional level of the mRNA by adding poly (A/T) tails at the 3’-UTR of the *sldAB* were discussed. It was revealed that an artificial poly (A/T) tail was proposed to slow down the mRNA degradation process in bacteria and the high *sldAB* expression levels were achieved. This well demonstrated the importance of the mRNA stability on the gene expression, which should be considered in our late study.

**Table 1:** Enzyme activities and relative transcriptional levels of the membrane-bound SLDH in *G. oxydans* strains

Strains	Specific SLDH activity (U/mg protein)	Relative transcriptional levels of *sldAB* gene
*G. oxydans* ZJU2	0.75 ± 0.02	1.03 ± 0.02 1.02 ± 0.03 4.12 ± 0.04
*G. oxydans* ZJU2/pBB5-P_0169_	0.74 ± 0.01	
*G. oxydans* ZJU2/pBB5-P_0169_-*sldAB*	2.55 ± 0.04	

### Batch fermentation by *sldAB* overexpression strain

**Fig. 3:**
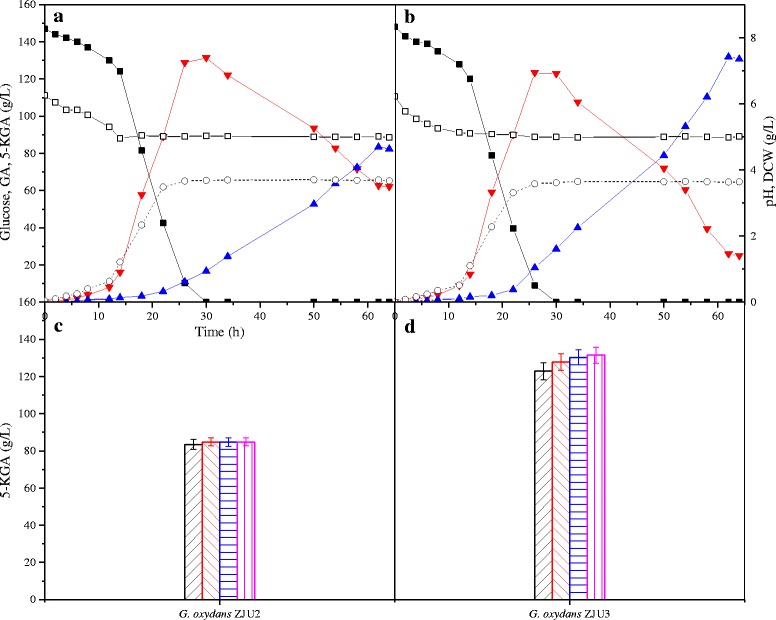
Time-course of the oxidative fermentation in a 15-L fermentation tank and coenzyme PQQ complement study. **a**, **c** *G. oxydans* ZJU2, **b**, **d** *G. oxydans* ZJU3 strains. ■ Glucose,  GA,  5-KGA, ○ DCW, □ pH value,  control,  100 μg/L,  200 μg/L,  500 μg/L

The batch fermentation by the recombinant *G. oxydans* ZJU3 strain was performed on a 15 L agitation tank under DOT rich condition. The results demonstrated that all tested strains had the similar trend of glucose consumption rate and cell growth (Fig. 3). The glucose was rapidly oxidized and exhausted at 30 h, while about 130 ± 1.76 g/L of GA was accumulated over the same time frame. The reference strain, *G. oxydans* ZJU2, accumulated 82.48 ± 1.10 g/L 5-KGA in the broth, but the residual GA was quite high at about 62.07 ± 1.04 g/L (Fig. 3a). In the fermentation process using recombinant *G. oxydans* ZJU3, 122.48 ± 0.41 g/L of 5-KGA with the productivity of 1.92 g/(L · h) were obtained at 64 h (Fig. 3b). Compared with the reference strain, the titer of 5-KGA was increased 48.99 % and the residual GA was 24.95 ± 0.76 g/L, decreased 59.80 %.

However, the DCW of the tested strains was about 3.58 ± 0.13 g/L, which was lower than other bacteria. In recent study, DNA microarray analysis and ^13^C metabolic flux analysis (^13^C -MFA) are used to characterize the two growth phases of *G. oxydans* in the presence of glucose [25]. In the first growth phase, 90 % of the glucose is oxidized by the mGDH to GA, accompanied by reasonable growth, high demand for oxygen and a low formation of CO_2_. The onset of phase II results in reduced biomass and demand for oxygen while the GA was oxidized in the periplasm to 5-KGA (Fig. 3). Thus, only a small percentage of the carbon source is taken up by cell, resulting in modest cell growth and poor cell yield. *G. oxydans* IFO3293 and *G. oxydans* 621H cells cultivated on glucose medium provids a cell yield of 0.09 g _cdw_/g _glucose_ [28, 29]. In comparison to these values, *E. coli* reaches a value of 0.49 g _cdw_/g _glucose_ [30], and *Bacillus subtilis* reaches a yield of 0.32 g _cdw_/g _glucose_ [31]. In phase I and II, the cytoplasmic sugar catabolism proceeded predominantly via the PPP, particularly in phase II [28].

During the glucose metabolism by *G. oxydans*, the mGDH and SLDH serve as the main enzymes, and PQQ is the cofactor. The PQQ supplementation experiments showed that the control strain *G. oxydans* ZJU2 was not significantly influenced by the exogenous PQQ (Fig. 3c), but the *G. oxydans* ZJU3 strain showed the respond to PQQ supplementation in a similar manner, achieving the highest 5-KGA production when the added PQQ was up to 500 μg/L (Fig. 3d). A total of 131.92 ± 2.11 g/L of 5-KGA was produced by *G. oxydans* ZJU3. These results indicated that the addition of PQQ could enhance the production of 5-KGA in the SLDH overexpression strain, which was in agreement with a recent report that the overexpression of PQQ-dependent dehydrogenases could lead to imbalance between coenzyme PQQ level and the corresponding quinoproteins [24].

### Enhanced PQQ biosynthesis to improve 5-KGA production

To further augment the 5-KGA production, the PQQ biosynthesis gene, *pqqABCDE*, was engineered and the biomass growth, PQQ concentration and 5-KGA production were shown in Table 2. In *G. oxydans* ZJU4, overexpression of *pqqABCDE* gene cluster under the control of P_0169_ promoter led to the excretion of 674.82 ± 4.12 μg/L of PQQ into the supernatant, which was enhanced by 383.53 % compared with that by the parent strain while the 5-KGA concentration was 131.76 ± 1.89 g/L, an increase of 7.58 %. This also confirmed the results from the PQQ supplement experiment (Fig. 3) that the coenzyme PQQ was the key factor driving the 5-KGA production. In *G. oxydans* ZJU5, simultaneous expression of the PQQ gene cluster and an associated *tldD* gene produced 757.83 ± 2.43 μg/L of PQQ, which was increased by 12.30 % compared with that from *G. oxydans* ZJU4. These results were consistent with the literature finding that the *tldD* gene was related to the PQQ biosynthesis [32]. The 5-KGA production by *G. oxydans* ZJU5 was 134.88 ± 2.16 g/L. In addition, the cell growth of *G. oxydans* ZJU5 was comparable to that of *G. oxydans* ZJU4. This finding implied that *tldD* gene expression did not affect the cell growth.

**Table 2:** Effects of overexpression *pqqABCDE* cluster and *tldD* genes in *G. oxydans* strains

Strains	Max. DCW (g/L)	PQQ concentration (μg/L)	5-KGA concentration (g/L)
*G. oxydans* ZJU3	3.58 ± 0.13	139.56 ± 1.87	122.48 ± 0.41
*G. oxydans* ZJU3/pUCpr	3.39 ± 0.09	137.74 ± 2.24	118.89 ± 1.28
*G. oxydans* ZJU3/pUCpr-T1	3.41 ± 0.11	674.82 ± 4.12	131.76 ± 1.89
*G. oxydans* ZJU3/pUCpr-T2	3.40 ± 0.08	757.83 ± 2.43	134.88 ± 2.16

Overexpression of metabolic pathways involving redox reactions may lead to cofactor imbalances, thus impairing the yield of products. Cofactor engineering approaches are often adopted to compensate for imbalance of cofactors to improve product biosynthesis [33], for example, manipulating the availability of intracellular NADH [34] and NADPH [35]. In *G. oxydans*, quinoproteins and their cofactor PQQ can catalyze the oxidation of substrates by PQQ regulated electron transfer in the respiratory chain. However, the biosynthesis of quinoproteins and their cofactor PQQ are usually independent [24]. Therefore, the PQQ gene cluster was overexpressed in *Gluconobacter oxydans* WSH-003 [23] and *Ketogulonigenium vulgare* [24], which demonstrated to improve 2-keto-L-gulonic acid (2-KLG) production by cofactor engineering. These advance significantly facilitated the development of the efficient strains to produce 5-KGA.

Notably, it was reported that disruption of *tldD* gene in *G. oxydans* led to a drop of PQQ excretion below the detection limit and a decrease in cell growth, indicating that the related *tldD* gene was essential for PQQ biosynthesis [32]. The TldD protein of *G. oxydans* 621H, related to the *E. coli* TldD, is a peptidase involved in processing of small peptides. In other PQQ-producing bacteria, the peptidase-like protein PqqF is required for PQQ synthesis and has a similar function as the TldD in *G. oxydans* [32]. Therefore, our study demonstrated that overexpression of the *tldD* gene could increase PQQ level by 12.30 % in *G. oxydans* ZJU5 compared with that of *G. oxydans* ZJU4 (Table 2). This cofactor engineering was adopted to compensate the shortage of cofactors in the PQQ-dependent SLDH overexpression strain and to improve the 5-KGA production.

### The engineering of the respiratory chain

In an earlier study, the cytochrome *bo*_3_ oxidase (*cyoBACD*, GOX1911-1914) was found to be the main component for proton extrusion via the respiratory chain in *G. oxydans* 621H [25]. To enhance the respiratory chain activity and the 5-KGA production, the cytochrome *bo*_3_ oxidase was engineered. The *cyoBACD* genes were fused into the PQQ overexpression plasmid (pUCpr-T1 and pUCpr-T2) under the P_0169_ promoter control, generating pUCpr-T3 and pUCpr-T4. The corresponding plasmids were electrotransferred into *G. oxydans* ZJU3, resulting in *G. oxydans* ZJU6 and *G. oxydans* ZJU7, respectively.

**Fig. 4:**
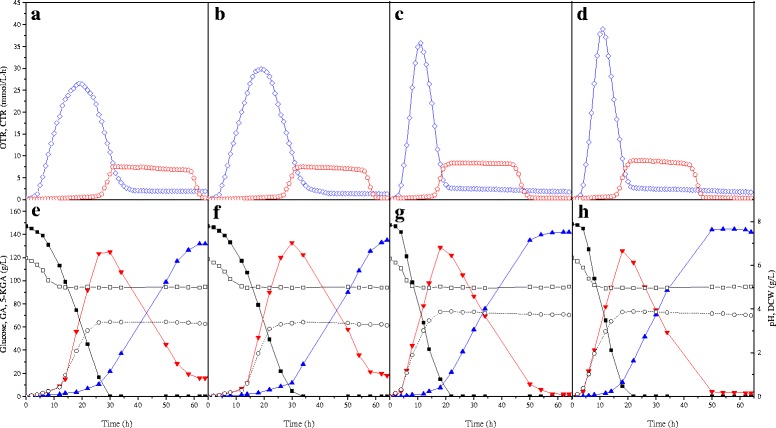
The OTR and CTR value and the glucose metabolism. **a**, **e** *G. oxydans* ZJU4, **b**, **f** *G. oxydans* ZJU5, **c**, (g) *G. oxydans* ZJU6, **d**, **h** *G. oxydans* ZJU7. ■ Glucose,  GA,  5-KGA, ○ DCW, □ pH value,  OTR, and  CTR

The batch fermentations of *G. oxydans* ZJU4, *G. oxydans* ZJU5, *G. oxydans* ZJU6, and *G. oxydans* ZJU7, under an excess of oxygen, showed that all recombinant *G. oxydans* strains reached their maximal OTR. The time points of maximal OTR correlated with the time points at which about 60 % glucose had been predominantly oxidized to GA in the periplasm (Fig. 4). *G. oxydans* ZJU4 and *G. oxydans* ZJU5 reached their maximal OTR of 26.52 mmol/L · h and 29.85 mmol/L · h, respectively at 19 h. After complete consumption of glucose, the OTR decreased to 1.5 to 1.8 mmol/L · h. The specific maximal CO_2_ production rate (CTR) of both strains was 7.5 mmol/L · h at 32 h, then it began to decrease (Fig. 4a, b). The CTR decreased below 5.0 mmol/L · h at 57 h for the *G. oxydans* ZJU5 strain, but it decreased below this level at 60 h for *G. oxydans* ZJU4. This meant that the *tldD* gene overexpression could increase the PQQ biosynthesis and the oxidation rate of SLDH. The situations of *G. oxydans* ZJU6 and *G. oxydans* ZJU7 were remarkable different with those of *G. oxydans* ZJU4 and *G. oxydans* ZJU5, where 141.86 ± 2.89 g/L and 144.52 ± 2.94 g/L 5-KGA was accumulated, respectively. The glucose oxidation rate was accelerated, and glucose was exhausted at 20 h. The maximal OTR of 35.83 mmol/L · h and 38.97 mmol/L · h were achieved at 11 h, respectively. After 22 h, the CTRs of ZJU6 and ZJU7 reached the maximal values of 8.38 mmol/L · h and 8.98 mmol/L · h, respectively. The CTR of *G. oxydans* ZJU7 decreased to 0.5 mmol/L · h at 47 h, which was 13 h and 3 h short than those of *G. oxydans* ZJU5 and *G. oxydans* ZJU6, respectively (Fig. 4c, d). This indicated that the oxidation of GA to 5-KGA by *G. oxydans* ZJU7 had completely finished at 47 h.

Using the constructed *G. oxydans* strains, the H^+^/O ratio and terminal ubiquinol *bo*_3_ oxidase activity were also measured when the cells were in the logarithmic growth phase (DCW of 1.5). The results were shown in Table 3. The average H^+^/O ratio and terminal ubiquinol oxidase activity of the control strain *G. oxydans* ZJU3 were 1.24 ± 0.11 and 0.31 ± 0.04 μmol/min · mg, respectively. The *G. oxydans* ZJU4 and *G. oxydans* ZJU5 had an H^+^/O ratio and terminal ubiquinol oxidase activity comparable to those of the control strain, implying the mutant growth was not impaired under conditions of PQQ cluster overexpression. However, the recombinant *G. oxydans* ZJU6 and *G. oxydans* ZJU7 showed a 64 % increase of the H^+^/O ratio (1.95 ± 0.23 and 2.01 ± 0.16) and 1.5-fold of the ubiquinol oxidase activity (0.78 ± 0.05 μmol/min · mg and 0.80 ± 0.06 μmol/min · mg) compared with the control strain, respectively. A total of 141.86 ± 2.89 g/L and 144.52 ± 2.94 g/L 5-KGA was accumulated by *G. oxydans* ZJU6 and *G. oxydans* ZJU7, respectively. This indicated the expression of ubiquinol *bo*_3_ oxidase could enhance respiratory proton translocation and increase the 5-KGA production in *G. oxydans*.

**Table 3:** H^+^/O ratio and ubiquinol oxidase activity of recombinant *G. oxydans* strains

Strains	H^+^/O ratio^*a*^ (No. of experiments)	Ubiquinol oxidase activity (μmol/min · mg)	5-KGA production (g/L)
*G. oxydans* ZJU3	1.21 ± 0.11 (8)	0.31 ± 0.04	122.48 ± 0.41
*G. oxydans* ZJU4	1.19 ± 0.10 (8)	0.32 ± 0.05	131.76 ± 1.89
*G. oxydans* ZJU5	1.20 ± 0.11 (8)	0.30 ± 0.11	134.88 ± 2.16
*G. oxydans* ZJU6	1.95 ± 0.23 (8)	0.78 ± 0.05	141.86 ± 2.89
*G. oxydans* ZJU7	2.01 ± 0.16 (8)	0.80 ± 0.06	144.52 ± 2.94

^*a*^the H^+^/O measured by the oxygen pulse method

*G. oxydans* possesses a branched respiratory chain consisting of two terminal ubiquinol oxidases, cytochrome *bo*_3_ oxidase and cytochrome *bd* oxidase (*cydAB*, GOX0278-0279) (Fig. 1). The genome sequence also revealed genes for a cytochrome *bc*_1_ complex (*qrcABC*, GOX0565-0567) and a soluble cytochrome *c* (GOX0258) [10]. The absence of cytochrome *bd* oxidase did not affect the cell growth or proton extrusion via the respiratory chain, whereas absence of the genes encoding cytochrome *bo*_3_ oxidase caused a severe growth defect [25]. Plasmid-based overproduction of cytochrome *bo*_3_ oxidase under the P_0169_ promoter control increased the respiration-driven proton extrusion by 66.7 % in *G. oxydans* ZJU6 and *G. oxydans* ZJU7, compared with the control strains (Table 3). The increase in the H^+^/O ratio might be owing to the presence of more *bo*_3_ quinol oxidase and its proposed high oxygen affinity, which should favor oxygen reduction under the experimental conditions of an oxygen pulse [25]. The H^+^/O ratio for the reference strain, *G. oxydans* ZJU3, was measured as 1.21 ± 0.11. In the literature, H^+^/O ratios reported for *E. coli* vary between 3.4 and 4.5 [36, 37], which was more than 2-fold higher than that of the *G. oxydans* ZJU3 strain. A major difference of the respiratory chain between the two species is the lack of the multisubunit proton-pumping NADH dehydrogenase I (NDH-I) in the *G. oxydans* [10, 25]. However, NDH-I is preferentially synthesized during anaerobic growth in the presence of alternate electron acceptors [38]. Therefore, what extent NDH-I contributes to the H^+^/O ratio is unclear. As the *bd* oxidase was not relevant for proton translocation, an H^+^/O ration of 4 might be assumed for *G. oxydans* [39]. However, our results were much lower than a value of 4. Richhardt explained that cytochrome *bo*_3_ oxidase might not function as a primary proton pump but as a Na^+^ pump and the respiratory chain could involve a reverse electron transfer coupled to an influx of protons [25].

Interestingly, an increased 15.48 % cell yield was observed when the *bo*_3_ oxidase gene was overexpressed (Fig. 4 g, h). The possibility of an increased *bo*_3_ oxidase level in *G. oxydans* could cause a shift of the electron flux from the non-proton pumping *bd* oxidase to the proton pumping *bo*_3_ oxidase [25]. Another explanation for the increased cell growth is the assumption that the activity of the *bo*_3_ oxidase is limited and can be increased by its overproduction. This was confirmed in the experiments that the ubiquinol oxidase activity increased 2.5-fold (Table 3) and the OTR enhanced 9.12 mmol/L · h (Fig. 4) by overexpressed the *cyoBACD* genes, indicating that cytochrome *bo*_3_ quinol oxidase played the positive role in the cell growth and substrate oxidation. Recently, a limitation of oxygen consumption by the activity of the terminal oxidase was reported after overexpressing the membrane-bound glucose dehydrogenase (mGDH, GOX0265) [40]. This implied that the activity of *bo*_3_ quinol oxidase might be high enough to satisfy the increased capacity for oxygen reduction.

### Process optimization and fed-batch fermentation

**Fig. 5:**
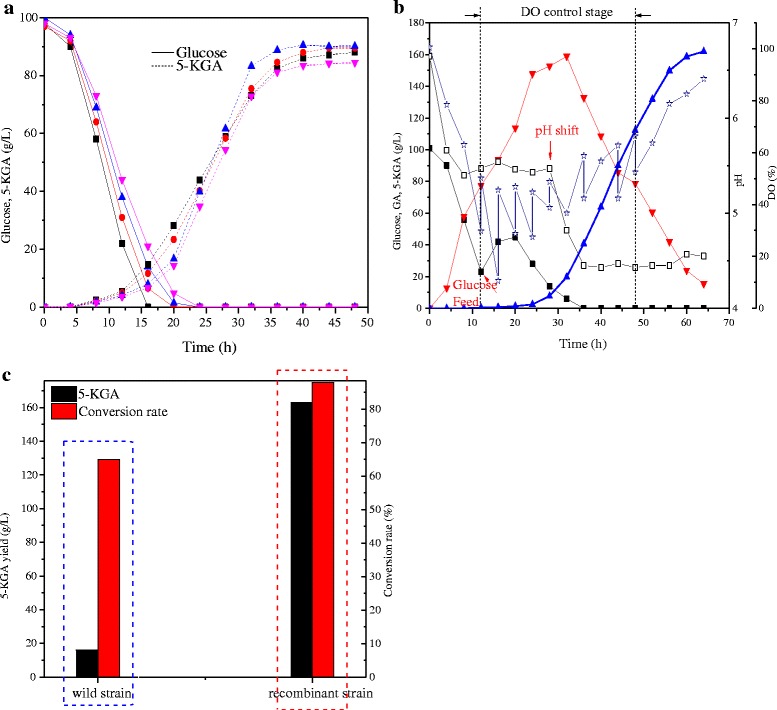
**a** Effect of pH on the glucose consumption and 5-KGA production by *G. oxydans* ZJU7. ■ pH 5.5,  pH 5.0,  pH 4.5,  pH 4.0. **b** The glucose fed-batch fermentation by *G. oxydans* ZJU7 under DO control and pH shift condition. ■ Glucose,  GA,  5-KGA, □ pH value and ☆ DO. **c** The 5-KGA production and conversion rate between *G. oxydans* ZJU7 and wild-type strains. black-bar, 5-KGA, red-bar, conversion rate

The constructed *G. oxydans* ZJU7 showed good potential for 5-KGA production. To fulfill the requirement of industrial production of 5-KGA, the fermentation process was optimized, including the two-stage pH control, DO control and glucose feed. As shown in Fig. 5a, the glucose was oxidized faster and exhausted at 14 h when the pH was controlled at 5.5. This result was consistent with the previous results where a pH of 5.5 was suitable for the activity of membrane-bound glucose dehydrogenase [41]. However, a pH of 4.5 was more suitable for synthesis of 5-KGA by SLDH. In the early study, the pH of the culture medium was an important factor for selective production of 5-KGA by *G. suboxydans* IFO 12528, in which an 87 % glucose conversion rate could be achieved by controlling the medium pH in a range of 3.5-4.0 [20]. According to the experimental results, we have established a two-stage pH control strategy, whereas the pH is controlled as 5.5 in the first stage to let the glucose oxidation and cell growth, then it is shifted to 4.5, facilitating 5-KGA formation.

In its natural habitat, *G. oxydans* is likely subjected to low-oxygen stress conditions because of the rapid oxygen consumption as a result of its own metabolism. The OTR and CTR were previously investigated during the 5-KGA production, in which the high OTR was observed when the glucose was oxidized to GA in the periplasm (Fig. 4a, b, c, d). It was reported that oxygen limitation could cause expression changes of 486 genes, representing 20 % of the chromosomal genes [42]. Therefore, during this stage, it must ensure the adequate DOT above 20 % by increasing the agitation speed. The onset of 5-KGA formation decreased the demand for DOT but increased the CTR (Fig. 4a, b, c, d). Hence, to improve the cost performance of the industrial production of 5-KGA, DOT was continuously controlled by adjusting the agitation speed according to OTR and CTR.

Additionally, to achieve the hyper-production of 5-KGA by the recombinant *G. oxydans* ZJU7 strain, the glucose fed-batch fermentation was carried out under the DOT and pH control. A glucose feed of about 1400 g was started at 12 h when the glucose concentration was below 40 g/L and was used to maintain the glucose at 40 ~ 50 g/L (Fig. 5b). The GA quickly accumulated during this stage. When the glucose concentration dropped to 14 g/L at 28 h, the 5-KGA began to quickly be formed and the pH shifted from 5.5 to 4.5. Concurrently, the DOT was controlled above 20 %. Using glucose fed-batch fermentation, DOT control, and a pH shift strategy, the 5-KGA titer was increased to 162 ± 2.12 g/L with the 2.53 g/(L · h) productivity by the recombinant *G. oxydans* ZJU7 strain, which was increased by 10-fold compared to the wild-type strain (Fig. 5c). This indicated that the PQQ overexpression and respiratory chain modification could efficiently enhance 5-KGA accumulation. Furthermore, the problem of residual GA was well solved, and the final concentration of GA was reduced to 15.38 g/L, which should decrease the separation difficulty. These results illustrated that the supplement of co-enzyme to the membrane-bound dehydrogenases was increased by overexpression of the PQQ cluster genes. The efficiency of electron transfer to O_2_ was enhanced by overexpression of the genes for the *bo*_3_ oxidase, which increased the activity of respiratory chain.

## Conclusion

Bacteria of the genus *Gluconobacter* exhibit so-called oxidative fermentation, or incomplete oxidation, a highly unusual metabolic feature that has been exploited industrially for decades in the production of vitamin C, GA and dihydroxyacetone [43]. In this study, the role of the combinatorial metabolic engineering of the industrial *G. oxydans* for the boosting of 5-KGA accumulation was investigated. In summary, the *sldAB* was overexpressed under the selected P_0169_ promoter. In addition, the PQQ gene cluster and terminal ubiquinol *bo*_3_ oxidase were fused overexpression to strengthen the glucose oxidation. Under the optimized culture conditions of the fed-batch process, the combinatorial approaches collectively increased the 5-KGA titer 10-fold, reached 162 ± 2.12 g/L. The results showed great potential for optimizing the current producer strain of *G. oxydans* used in industrial biotechnology, which presents the first report of 5-KGA production by combinatorial metabolic engineering approaches in *G. oxydans*. We envision that these approaches could provide framework for devising engineering strategies to improve the production of biochemicals in *G. oxydans*. Nevertheless, some issues are still worth further study, for example, how many copies of *pqq* cluster and *bo*_3_ oxidase genes overexpression can be matched the quinoproteins requirements, especially in the quinoproteins overexpression strains. The genome sequence has revealed that the TCA cycle is incomplete as genes for succinate dehydrogenase and succinyl CoA synthetase were absent [10]. To develop more robust strains, the strategy involving direct repair of this defective metabolic pathway by genomic integration of heterologous genes should be investigated, while considering the *G. oxydans* as a broadly applicable host for oxidative industrial bioconversions.

## Methods

### Bacterial strains, plasmids, and media

The bacterial strains, and plasmids used in this study are listed in Table 4. *Escherichia coli* strains were cultivated in Luria–Bertani (LB) medium at 37 °C. The 50 μg/mL gentamicin or 100 μg/mL ampicillin were used whenever required. Agar (1.5 %) was added to obtain solid media. *Gluconobacter oxydans* DSM2343 strains were cultivated on mannitol medium (MP) containing 5 g/L yeast extract, 3 g/L peptone, and 25 g/L mannitol at 30 °C. For *G. oxydans* possesses a natural resistance against cefoxitin, thus, as a precaution to prevent bacterial contamination, 50 μg/mL cefoxitin was added.

**Table 4:** Bacterial strains and plasmids used in this work

	Properties	Source
Strains		
*E. coli* DH5α	*F* ^−^, *endA1*, *hsdR17* (rk-mk-),*supE*44, *thi*1, *recA*1, *gyrA*, (Nalr), *relA*1, D(*lacZYAargF*), U169, and F80*lacZ*DM15	Invitrogen
*G. oxydans* DSM2343	Wild-type, Cef^R^	DSMZ^a^
*G. oxydans* ZJU2	Gluconate 2-dehydrogenase and pyruvate decarboxylase deletion strain derived from *G. oxydans* DSM2343, Cef^R^	[21]
*G. oxydans* ZJU3	*G. oxydans* ZJU2 harboring pBB5-P_0169_-*sldAB*, Cef^R^, Gm^R^	This work
*G. oxydans* ZJU4	*G. oxydans* ZJU3 harboring pUCpr-T1, Cef^R^, Gm^R^, Amp^R^	This work
*G. oxydans* ZJU5	*G. oxydans* ZJU3 harboring pUCpr-T2, Cef^R^, Gm^R^, Amp^R^	This work
*G. oxydans* ZJU6	*G. oxydans* ZJU3 harboring pUCpr-T3, Cef^R^, Gm^R^, Amp^R^	This work
*G. oxydans* ZJU7	*G. oxydans* ZJU3 harboring pUCpr-T4, Cef^R^, Gm^R^, Amp^R^	This work
Plasmids		
pUC19	Cloning vector, ColE1 *ori*, Amp^R^	Invitrogen
pET28 (a)-GFP	*gpf* gene expressed vector, Km^R^	Laboratory preservation
pBBR1MCS-5	Broad-host-range (bhr) expression vector, Gm^R^	[45]
pBB5-P_tufB_	Insert P_tufB_ promoter vector derived from pBBR1MCS-5, Gm^R^	This work
pBB5-P_0264_	Insert P_0264_ promoter vector derived from pBBR1MCS-5, Gm^R^	This work
pBB5-P_0452_	Insert P_0452_ promoter vector derived from pBBR1MCS-5, Gm^R^	This work
pBB5-P_0169_	Insert P_0169_ promoter vector derived from pBBR1MCS-5, Gm^R^	This work
pBB5-P_0169_-*sldAB*	*sldAB* gene overexpression vector derived from pBBR1MCS-5, inserted P_0169_ promoter, Gm^R^	This work
pUCpr	Constructed expression vector derived from pUC19, *par-rep*, Amp^R^	This work
pUCpr-T1	pUCpr-P_0169_-*pqqABCDE* vector derived from pUCpr, Amp^R^	This work
pUCpr-T2	pUCpr-P_0169_-*pqqABCDE*-*tldD* vector derived from pUCpr, Amp^R^	This work
pUCpr-T3	pUCpr-P_0169_-*pqqABCDE*-P_0169_-*cyoBACD* vector derived from pUCpr, Amp^R^	This work
pUCpr-T4	pUCpr-P_0169_-*pqqABCDE*-*tldD*-P_0169_-*cyoBACD* vector derived from pUCpr, Amp^R^	This work

^a^DSMZ, Deutsche Sammlung von Mikroorganismen und Zellkulturen, Braunschweig, Germany

### Construction of shuttle vector

A shuttle vector pUCpr, compatible to the broad-host-plasmid pBBR1MCS5, was constructed. The 2446-bp *par*-*rep* gene fragment of cryptic plasmid pGOX3 from *G. oxydans* DSM2343 was amplified with primers pr_PstI_F / pr_SalI_R (Table 5). The sequenced PCR product was digested and inserted into the *Pst*I/*Sal*I site of the pUC19, resulting in pUCpr [44].

**Table 5:** The oligonucleotides primers used in this work

Primer	Sequence (5’ → 3’)	Usage
pr_PstI_F	AACTGCAGgtttatcggccgttgaatat	Amplify the *par-rep* gene
pr_SalI_R	ACGCGTCGACggtgtttaaacagtgttacggt	
0169_SacI_F	ATAGAGCTCtgaaagcggctggcgcgt	Amplify the 5’-UTR of GOX0169 promoter
0169_XbaI_R	GCTCTAGAgcggaaggcgttataccctga	
0264_SacI_F	ATAGAGCTCgttgcgcctgaatgagagg	Amplify the 5’-UTR of GOX0264 promoter
0264_XbaI_R	GCTCTAGAttcggtctccctcgccgtaa	
0452_SacI_F	ATAGAGCTCggcttcgtggtgaacgcc	Amplify the 5’-UTR of GOX0452 promoter
0452_XbaI_R	GCTCTAGAtagtgacattccagcttggg	
tufB_ SacI_F	ATAGAGCTCcgatggtaagaaatccactgc	Amplify the *tufB* promoter
tufB_ XbaI_R	ATATCTAGAccaaaaccccgctccacc	
GFP_XbaI_F	ATATCTAGAatggtgagcaagggc	Amplify the *gfp* reported gene
GFP_HindIII_R	CCCAAGCTTctacttgtacagctc	
SLDH_XbaI_F	GCTCTAGAggactttcagttctggaggctttcacca	Amplify the *sldAB* gene
SLDH_EcoRI_R	CGGAATTCtcccacccgaaaaatggaaaaaacg	
ADD_0169_F	acactgtttaaacaccgtgaaagcggctggcgc	Amplify the fuse fragments
pQQ_Fuse0169_R	acatccgcgcggaaggcgttatac	
pQQ_Fuse0169_F	ccttccgcgcggatgttcagg	
tldD_FusepQQ_R	ccggctagaagatggcctctc	
tldD_FusepQQ_F	gccatcttctagccggtctgttc	
0169_FusetldD_R	ctttcaggatcttcttcatg	
0169_FusetldD_F	tcgcgactgaaagcggctggc	
ADD_0169_R	cggtacccggggatcctgcggaaggcgttatac	
cyoBACD_XbaI_F	CGATTCTAGAactactgcaagccggaacgg	Amplify the terminal cytochrome *bo*_3_ oxidase
cyoBACD_SacI_R	ACTGGAGCTCaagggctggcaggatttctc	
RT16S_F	gcggttgttacagtcagatg	-
RT16S_R	gcctcagcgtcagtatcg	-
RTsldh_F	atcatgccgaccaagcgtggc	-
RTsldh_R	cgtcggcgaacgcggatcg	-

^*a*^The capital and underlined sequences indicate the restriction enzyme sites

### Promoter selection

Four different promoters from *G. oxydans* DSM2343 were carefully selected. A putative promoter sequence, P_0169_ [22], the promoter of elongation factor *Tu*, P_tufB_ [26], and ribosomal proteins L35 and L13, P_0264_ and P_0452_ [27], were amplified by PCR with primers as listed in Table 5. The resulting DNA fragments P_tufB_, P_0264_, P_0452_ and P_0169_ were digested with restriction enzymes *Sac*I and *Xba*I, then were ligated into pBBR1MCS-5 [45], generated the vector pBB5-P_tufB_, pBB5-P_0264_, pBB5-P_0452_ and pBB5-P_0169_, respectively. The report gene *gfp* was amplified from the cloning vector pET28 (a)-GFP. The resulting product was digested with *Xba*I and *Hind*III, and then cloned into the *Xba*I and *Hind*III site of pBB5-P_tufB_, pBB5-P_0264_, pBB5-P_0452_ and pBB5-P_0169_ to generate the corresponding promoter strength reporter plasmids.

The reporter plasmids were first transformed into *E. coli* DH5α, analyzed for the correct insert by DNA sequencing and then transformed into *G. oxydans* DSM2343 by electroporation (2000 V, 200 Ω and 25 μF) in a 2-mm cuvette using a Gene Pulser II (Bio-Rad, München, Germany) as described previously [46]. The transformants were selected by cefoxitin and gentamicin. The whole cell fluorescence intensity (RFU/OD, the relative fluorescence unit divided by the corresponding cell density) was measured [22]. Cells were harvested and washed twice with KPB buffer (pH 7.0), and then photographed by using a confocal laser scanning microscope. The cell density was measured by the absorbance at 600 nm using the spectrophotometer (UVmini-1240, SHIMADZU^®^), which determined the strengths of the different promoters.

### Overexpression of *sldAB* gene in *G. oxydans* ZJU2

Based on the bioinformatics analysis of *G. oxydans* 621H genome sequence [10], SLDH_XbaI_F / SLDH_EcoRI_R primers were designed and the open reading frames (ORFs) of SLDH (*sldAB*, GOX0854-0855) was PCR-amplified with the primers. The genomic DNA of *G. oxydans* DSM 2343 was used as a template. The sequenced amplicon was digested with the restriction endonucleases *Xba*I and *EcoR*I and cloned into pBB5-P_0169_ vector restricted with the same enzymes, resulting in plasmids pBB5-P_0169_-*sldAB*. This plasmid and as a control pBB5-P_0169_ vector were transferred into the desired *G. oxydans* ZJU2 strain by electroporation and were selected for a gentamycin-resistant phenotype. The correct strain *G. oxydans* ZJU2/pBB5-P_0169_-*sldAB* was named *G. oxydans* ZJU3.

### Co-expression of the PQQ biosynthesis genes and the terminal ubiquinol cytochrome *bo*_3_ oxidase genes in *G. oxydans* ZJU3

In the oxidation process by the membrane-bound SLDH, the PQQ serves as prosthetic groups [10, 15, 16], which transfer electrons to the respiratory chain [10]. To enhance the 5-KGA production, the genes encoding the cofactor PQQ and the terminal ubiquinol cytochrome *bo*_3_ oxidase of the respiratory chain were reinforced. To fulfill the experiments, the PQQ gene cluster (GOX0983-0987) and the related *tldD* gene (GOX1104) were cloned and the generated plasmids pUCpr-T1 and pUCpr-T2. The constructed plasmids were transferred into *G. oxydans* ZJU3, resulting the recombinant strains *G. oxydans* ZJU4 and *G. oxydans* ZJU5, respectively. The PQQ and terminal ubiquinol cytochrome *bo*_3_ oxidase fused expression plasmid was constructed by the *pEASY*-Uni seamless cloning and assembly kit CU101 (TransGen, China). The PCR-amplified promoter P_0169_, *pqqABCDE*, and *tldD* were cloned into the *Xba*I site of the shuttle vector pUCpr, which resulted in the plasmid pUCpr-T1-P_0169_ and pUCpr-T2-P_0169_. Then the *cyoBACD* (GOX1911-1914) was inserted the *Xba*I and *Kpn*I site of pUCpr-T1-P_0169_ and pUCpr-T2-P_0169_ to generate the fusion expressed plasmid pUCpr-T3 and pUCpr-T4, respectively. After verification of the accuracy of the plasmid by the sequencing, the fusion plasmid was transferred in the recombinant strain *G. oxydans* ZJU3 by electroporation [46] and selected for a gentamycin-kanamycin resistant phenotype, generated *G. oxydans* ZJU6 and *G. oxydans* ZJU7.

### Measurements of enzyme activity and protein concentration

For the purpose of preparation of the cell crude extract and membrane fraction, a single colony of the *G. oxydans* strain was pre-incubated in MP medium. The *G. oxydans* cells were harvested by centrifugation (10,000 × g, 5 min, 4 °C) and resuspended in 20 mL 50 mM sodium phosphate buffer (pH 6.0). The cells were disrupted with an ultrasonifier (JY92–2D, Xinzhi, NingBo) for 50 cycles (2000 w, 3 s sonication, 5 s pause) on ice. The cell debris were removed by centrifugation at 5500 × g for 20 min at 4 °C and the supernatant was used as the crude extract. For the preparation of membranes, the supernatant was centrifuged for 60 min at 180,000 × g at 4 °C. The resulting sediments were collected and resuspended into 50 mM sodium phosphate buffer (pH 6.0), and used as the membrane fraction.

Enzyme activities were determined using a spectrophotometer (Uvmini-1240 SHIMADZU^®^). Substrate-dependent changes of redox states of artificial electron acceptors (2, 6-dichlorophenolindophenol, DCPIP, Sigma) were determined at 600 nm and 30 °C. The basal reaction mixture contained 50 mM PBS pH 6.0, 0.25 mM DCPIP, and 0.325 mM phenazine methosulphate (PMS, Sigma), which was prepared and pre-warmed to 30 °C before the assay. Measurements were performed in a cuvette with a 1-cm light path containing a 0.8-mL basal reaction mixture and 10 μL enzyme pre-incubated at 30 °C for 5 min. The reaction was started by adding 20 μL of a 2.0 M gluconate solution. One unit of enzyme activity (U) was defined as the amount of enzyme that can catalyze the conversion of 1 μM DCPIP per min at 30 °C. The concentration of proteins was determined with the Pierce™ BCA assay kit.

### Quantitative real-time PCR (RT-PCR)

Cells were harvested at an OD_600_ of 2.5 at room temperature and immediately frozen in liquid nitrogen. Cells were then stored at −80 °C until RNA extraction. Total RNA was extracted with RNAiso™ Plus from Takara (Dalian, China) and treated with RNase-free DNase. Following chloroform extraction step, RNA was precipitated with isopropanol and the pellet washed twice in 75 % ethanol. After air-drying, RNA was resuspended in RNase-free water. The quantity of total RNA was verified using an Eppendorf Biophotometer (Eppendorf, Hamburg, Germany). The cDNA was synthesized from the total RNA using a PrimeScript RT Reagent Kit (Perfect Real Time) (Takara) according to the manufacturer’s protocol. The products were quantified via real-time PCR with StepOnePlus^TM^ Real-Time PCR System (Applied Biosystems, USA) using primer RTsldh_F/RTsldh_R. The 16S rRNA gene was used as internal standard based on the primer RT16S_F/RT16S_R.

### Characterization of respiration activity and H^+^/O measurements

Cultivation of *G. oxydans* strains was performed and the respiration activity was measured by the exhaust gas analysis system, such as the oxygen transfer rate (OTR), carbon dioxide transfer rate (CTR). The number of H^+^ moved upon respiration (H^+^/O ratio) is principally important for the efficiency of cellular ATP production. Hence the H^+^/O ratio of bacterial cells have been measured to evaluate the efficiency of the respiratory chain. The H^+^/O and terminal ubiquinol oxidase activity were measured using a method previously reported in the literature [36].

### Batch fermentations

Batch and fed-batch fermentations were conducted in a 15 L stirred tank bioreactor (Fus-D; Guoqiang Bioengineering Equipment Co., Ltd, Shanghai, China) with 9 L of the initial medium, which was composed of 0.41 g/L (NH_4_)_2_SO_4_, 0.1 g/L (NH_4_)_2_HPO_4_, 0.01 g/L MgSO_4_ · 7H_2_O, 3.0 g/L corn steep liquor paste and 100–150 g/L glucose (depending on the experiment) [19]. The CaCO_3_ (20 g/L) was sterilized separately and then added to the medium. The seed culture was prepared by inoculation of a single colony into a 5-mL MP medium tube, then into 200 mL of fresh seed medium in 500-mL flasks and cultivated on a rotary shaker at 220 rpm for 16 h. The seed culture (5 %, v/v) was then inoculated into the fermentation medium and the fermentation was carried out at 30 °C and pH was controlled at 5.0 by automatic addition of 5 M NaOH. In order to examine whether PQQ could enhance the 5-KGA production in engineered strains, different amount of PQQ (0 μg/L, 100 μg/L, 200 μg/L, 500 μg/L) was exogenously added to the mixed cultures at the beginning of the fermentation process, and the 5-KGA concentrations were measured after 64 h.

### Fed-batch fermentation

In this study, optimization of the pH condition was examined in a medium containing 100 g/L glucose as the initial substrate. The pH was controlled as 5.5, 5.0, 4.5, and 4.0 and the glucose and 5-KGA were detected. Hence, a two-stage pH control strategy was employed. In the first stage of glucose oxidized to GA, then shifted to 4.5 in the process of 5-KGA production. In further experiment, fed-batch culture was performed. When the fermentation was began, the initial volume was 6 L and the glucose concentration was 100 g/L, then 2 L of the feed medium containing 1400 g glucose was added when the concentration of glucose turned to 30 g/L and maintained between 30 and 40 g/L.

### Analysis

Samples were centrifuged at 12,000 g for 2 min, and the supernatant was passed through a 0.22 μm filter. The residual glucose concentration was determined by a bio-analyzer (SBA-40D, Shandong Academy of Sciences, China) after dilution to an appropriate concentration. The GA and 5-KGA in the fermentation broth were analyzed by high-performance liquid chromatography (HP1100, Agilent 1100 series) using a RSpak DE-613 column (Shodex, Japan), with 2 mM HClO_4_ as the mobile phase at a flow rate of 0.5 mL/min and a UV absorption of 210 nm. Acetic acid was detected by GC (Agilent 6820 series). The biomass dry cell weight was determined by applying membrane filtration. Before filtration, the CaCO_3_ was removed by reaction with HCl. The concentration of PQQ in the culture supernatants was measured according to the literature [24]. All experiments were performed in triplicate and the Origin 8.0 software package was used for statistical analysis. Analysis of variance was performed. Each data point represents the mean ± SD from triplicate experiments.

## Ethics approval

Not applicable.

## Consent for publication

Not applicable.

## Availability of data and materials

The Nucleic acids sequences supporting the conclusions of this article is available in the GenBank (National Center for Biotechnology Information) [http://www.ncbi.nlm.nih.gov/genbank].

Genome of *Gluconobacter oxydans* DSM2343 Accession: NC_006677.1 *par-rep* gene sequence from pGOX3 of *Gluconobacter oxydans* DSM2343 Accession: CP000006, pBBR1MCS5 plasmid Accession: U25061.

## Abbreviations used

2-KGA: 2-keto-D-gluconate
5-KGA: 5-keto-D-gluconate
DCPIP: 2, 6-dichlorophenolindophenol
DCW: dry cell weight
DOT: dissolved oxygen tension
GA: gluconic acid
PQQ: pyrroloquinoline quinone
